# Fluorescent proteins function as a prey attractant: experimental evidence from the hydromedusa *Olindias formosus* and other marine organisms

**DOI:** 10.1242/bio.012138

**Published:** 2015-07-31

**Authors:** Steven H. D. Haddock, Casey W. Dunn

**Affiliations:** 1 Monterey Bay Aquarium Research Institute (MBARI), 7700 Sandholdt Rd, Moss Landing, CA 95039-9644, USA; 2 Department of Ecology and Evolutionary Biology, Brown University, Box GW, 80 Waterman St, Providence, RI 02912, USA

**Keywords:** Fluorescent protein, *Olindias*, Prey attraction, GFP, Feeding behavior, Supernormal stimulus

## Abstract

Although proteins in the green fluorescent protein family (GFPs) have been discovered in a wide array of taxa, their ecological functions in these organisms remain unclear. Many hypothesized roles are related to modifying bioluminescence spectra or modulating the light regime for algal symbionts, but these do not explain the presence of GFPs in animals that are non-luminous and non-symbiotic. Other hypothesized functions are unrelated to the visual signals themselves, including stress responses and antioxidant roles, but these cannot explain the localization of fluorescence in particular structures on the animals. Here we tested the hypothesis that fluorescence might serve to attract prey. In laboratory experiments, the predator was the hydromedusa *Olindias formosus* (previously known as *O. formosa*), which has fluorescent and pigmented patches on the tips of its tentacles. The prey, juvenile rockfishes in the genus *Sebastes*, were significantly more attracted (*P*<1×10^−5^) to the medusa's tentacles under lighting conditions where fluorescence was excited and tentacle tips were visible above the background. The fish did not respond significantly when treatments did not include fluorescent structures or took place under yellow or white lights, which did not generate fluorescence visible above the ambient light. Furthermore, underwater observations of the behavior of fishes when presented with a brightly illuminated point showed a strong attraction to this visual stimulus. *In situ* observations also provided evidence for fluorescent lures as supernormal stimuli in several other marine animals, including the siphonophore *Rhizophysa eysenhardti*. Our results support the idea that fluorescent structures can serve as prey attractants, thus providing a potential function for GFPs and other fluorescent proteins in a diverse range of organisms.

## INTRODUCTION

Autofluorescence is a common phenomenon in the natural world, and a variety of molecules can re-emit absorbed photons as light of a longer wavelength. Natural fluorescent molecules include chlorophyll, phycobiliproteins, porphyrins, chitin, and green-fluorescent proteins (GFPs). Plants and algae, parrots, mantis shrimp, squids, penguins, and scorpions are some of the many organisms that are noted for their fluorescence (Arnold et al., 2002; Kloock, 2008; Mäthger and Denton, 2001; McGraw et al., 2007; Mazel et al., 2004). For nearly all of these cases, there is no experimental evidence to support an ecological function of the fluorescence, and much of the time – for example, fluorescent keratin in human fingernails – it may merely be a byproduct of the molecule's chemical structure, without functional significance.

In the family of GFPs, which are found in cnidarians, crustaceans, and chordates (Deheyn et al., 2007; Matz et al., 1999; Shagin et al., 2004; Wiedenmann et al., 2002), the fluorescent signal is so strong that the proteins are assumed to be present primarily because their fluorescence serves a role, even though for most GFP-bearing organisms the natural functions remain unclear. It is possible that GFPs serve a physiological function unrelated to their fluorescence (D'Angelo et al., 2008; Palmer et al., 2009), but their quantum efficiency and the morphological and spectral diversity of fluorescent structures that have evolved indicates that this is not the case. Suggested hypotheses relate to the modification of bioluminescence emission (Haddock and Case, 1999; Morin and Reynolds, 1969), or in the case of Anthozoans, modulation of the light environment of algal symbionts, either through photoprotection or photoehancement (Kawaguti, 1969; Salih et al., 2000; Schlichter et al., 1994). In an ecological context, absorptive pigments are only effective for achieving coloration when there are many wavelengths of light present in the environment. In the ocean, however, the range of wavelengths decreases rapidly with depth, until predominantly what we perceive as blue light remains. In such a monochromatic environment, differential absorption by pigments can only provide coloration in the form of variations in shades of blue (Johnsen, 2005; Marshall, 2000). In that same monochromatic habitat, however, fluorescent pigments provide a way to produce conspicuous or contrasting colors by converting the energy of blue photons into longer wavelengths of green, yellow, orange, and red. Thus the presence of fluorescent proteins can be an effective way to produce undersea coloration patterns, with the excitation coming from either ambient or bioluminescent light. These pigments are so long-lived and efficient that this vivid coloration comes at a relatively low metabolic cost (Leutenegger et al., 2007).

**Fig. 1.**
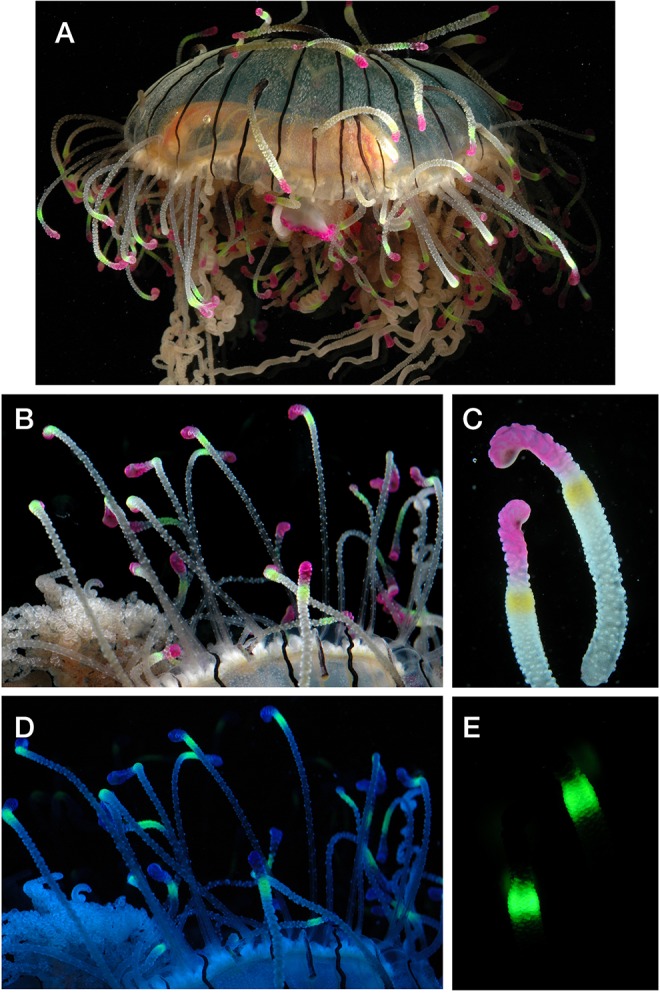
**Fluorescence of *Olindias.*** Photos of *O. formosus* in (A-C) white light and (D,E) under blue light, showing the fluorescence. Under white light (B) the fluorescence is excited, but is not distinct against the full-spectrum background illumination. (B,C) The tips of the tentacles have a pink chromoprotein which absorbs blue and green light, and thus appears dark in (D). Panel D is shown without a barrier filter, so the blue excitation has not been subtracted. Panel E shows the view with a long-pass filter so the blue-excitation is removed.

Among cnidarians, most of the animals which are known to have GFP either have algal symbionts (e.g. corals and anemones) or they are bioluminescent, including the well-studied species *Aequorea victoria* and the sea pansy *Renilla reniformis* (Prasher et al., 1992; Ward and Cormier, 1979). There are, however, many anthozoans and a few species of hydrozoans which have fluorescent structures without being bioluminescent (Matz et al., 1999). One such species is the limnomedusa *Olindias formosus* (Goto, 1903) (changed from *O. formosa* due to a gender mismatch between genus and species), which is found in shallow waters (<30 m deep) off the coast of Japan. It has a unique morphology wherein the tentacles grow up along the outside of bell, and terminate in green fluorescent and pink pigmented segments (Fig. 1). The life cycle of *O. formosus* was recently studied, and it was found that fluorescence is present even during early development of the polyp stage (Patry et al., 2014).

Purcell (1980) was among the first to suggest that siphonophore tentacles (Cnidaria, Hydrozoa) sometimes bore lures which were used for aggressive mimicry, and medusae have been suggested to use their tentacles in a similar manner (Larson, 1986; Courtney et al., 2015). Although these examples do not involve fluorescence, we have previously found siphonophores with lure-like tentilla that are either fluorescent (Pugh and Haddock, 2010) or bioluminescent (Haddock et al., 2005). Such observations led us to investigate the hypothesis that fluorescent proteins might serve to attract the attention of prey. This is important because the current suite of proposed functions for fluorescent proteins do not apply to many of the organisms that possess them (e.g. animals lacking symbionts and bioluminescence).

We conducted experiments using a non-luminous, non-symbiont-bearing hydromedusa as the predator and juvenile fishes as its potential natural prey. We also recorded *in situ* observations of fish behavior when presented with a conspicuous green spot of illumination, and gathered *in situ* and laboratory observations of fluorescence associated with prey-capture structures in a diverse array other marine animals.

## RESULTS

### Medusa morphology and spectroscopy

The predator *O. formosus* had green fluorescent bands near the tips of its upturned tentacles, with a blue-absorbing chromoprotein (appearing pink in white light) at the tentacle tip (Fig. 1).

Non-lethal laboratory feeding experiments were conducted with *Sebastes* spp. rockfish as prey and *O. formosus* medusae as the predator. Illumination of the medusa was alternated between blue, yellow, and white LEDs, which resulted in varying visual stimuli as described below.

**Fig. 2.**
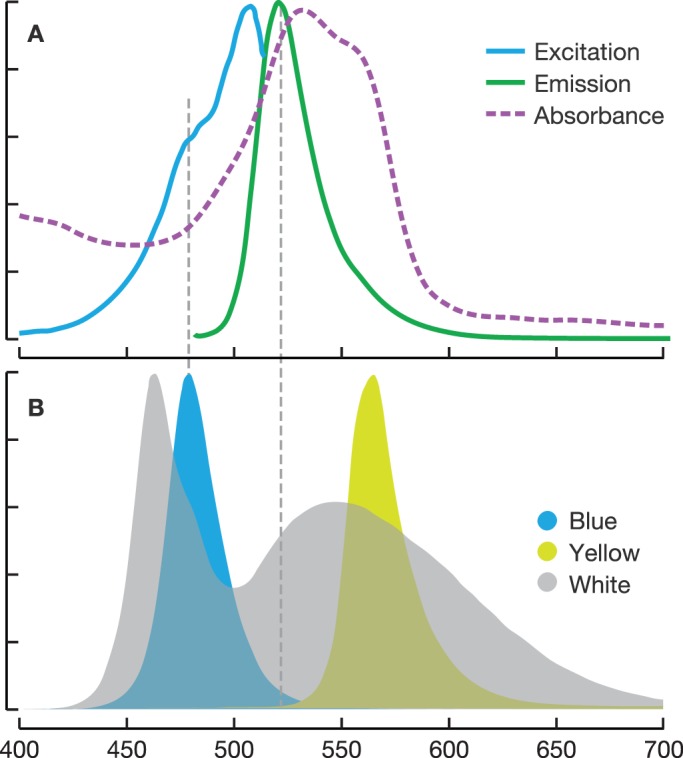
**LED spectra subset.** (A) Excitation (blue) and emission (green) of the green fluorescent protein and absorbance spectrum of the pink chromoprotein in tentacle tips of *O. formosus*. (B) LED emission spectra for the three treatments used in the experiment. Blue LED excites the fluorescent protein with minimal overlap with the emission spectrum (dashed grey line). Yellow LED is longer wavelength than the excitation spectrum of the fluorescence. X-axis, wavelength in nm.

The blue LED produced a maximum emission at 479 nm and the yellow LED at 566 nm (Fig. 2B). The blue LED emission overlapped with the excitation spectrum measured for *O. formosus* GFP (Fig. 2A), and thus was suitable to excite the green fluorescence of the tentacle tips. Blue light was also close to expected light field in the natural habitat of the medusa. The fluorescence emission of the tentacle tips was within the expected visual range of the *Sebastes* species we used as potential prey, and indeed most juvenile fishes have rods and cones sensitive to more than one wavelength extending beyond that range (Britt et al., 2001). In contrast, the yellow LED produced wavelengths longer than the excitation or emission spectrum of the fluorescent protein, and thus the medusa and its tentacles were only visible by their overall shape, with no distinct fluorescent features. The white LED produced two peaks, a sharp peak at 464 nm and a broader less intense peak at 548 nm (Fig. 2A). The short wavelengths of white light could potentially excite fluorescence, but it would not be conspicuous because the white LED also provided long-wavelength background illumination. Tentacle tips showed a peak fluorescent emission at 529 nm (Fig. 2B).

Absorbance of the pink chromoprotein region broadly covered the blue-green region (Fig. 2A, dashed line). This indicates that the chromophore, being non-fluorescent, would appear dark in a natural blue environment, potentially increasing the contrast with the adjacent fluorescent region of the tentacle. (See Fig. 1 for white light and blue-light views.)

### Prey attraction experiments

Medusae or mimic objects (as controls) were placed in one side of the tank, and fish were placed into the other side, with a clear barricade separating them. This barricade created a medusa partition occupying one fourth of the tank's width. The mimic (called the blobject) was a white translucent dome constructed out of a small inverted glass dish covered in Parafilm, imitating the size, shape, and color of the medusa, but without tentacles. An opaque barrier divided the tank in half, and this was removed at the beginning of each trial. Fish ‘interest’ in the medusa or blobject was quantified in two ways: (1) Time spent in the medusa half of the tank, and (2) number of attack strikes directed at the medusa.

#### Time spent

The time-spent data show a significantly elevated mean time spent in the region of interest (T_roi_) only for the blue light plus medusa treatment (*P*<0.04; Type I ANOVA) when compared to both blobject and no object (Table 1). Other blue light treatments, and yellow or white light showed no differences for T_roi_ for plus-medusa, no medusa, and blobject treatments.

**Table 1.**
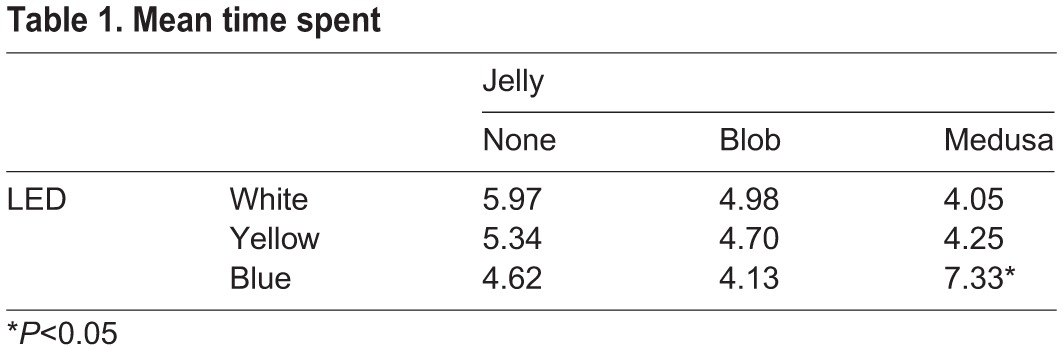
**Mean time spent**

#### Attack data

**Fig. 3.**
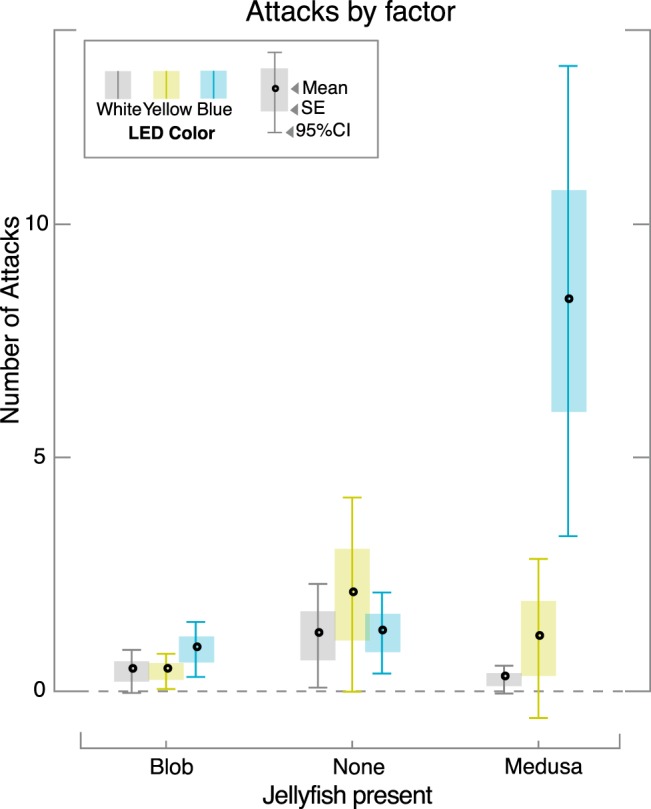
**Box plots of number of attacks.** Number of attacks plotted by the factor (medusa present or control conditions) and for each of three lighting schemes (color of bars). Box plots show mean (dot), standard error (shaded box height) and 95% confidence interval (whisker height). Significant differences in the number of attacks (*P*≪1×10^−5^) were obtained only for the treatment that included a live medusa with blue illumination. Attack behavior with the control objects and yellow or white lighting conditions were not significantly different from each other.

The number of times the fish attacked the clear barrier (N_a_) was also measured (Fig. 3; Table 2) and showed a significantly higher mean only for the medusa under blue illumination (Type I ANOVA, *P*<1×10^−5^). N_a_ was nearly 7 times higher when the medusa was present compared to either control. The other lighting schemes show no significant differences in N_a_ and, like T_roi_, even show a moderate decrease when the medusa is present.

**Table 2.**
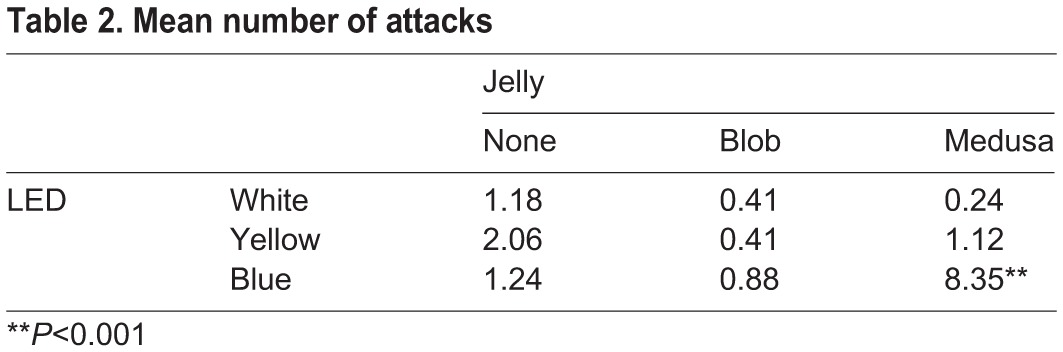
**Mean number of attacks**

#### Behavior during trials

Under the blue light+medusa treatment when the attack frequency was elevated, the fish behavior included quick, directed swimming motions, unlike the undirected swimming seen under control conditions. Occasionally attacks were made on the front glass of the tank, where a faint reflection may have been visible. There was also infrequent direct interest in the LEDs themselves through the surface of the water. This was shown by a vertical body orientation directly below the light and a body thrust up toward it. However both of these behaviors were rare enough not to affect the quantified trials.

#### *In-situ* responses of fish to fluorescence-like stimuli

**Fig. 4.**
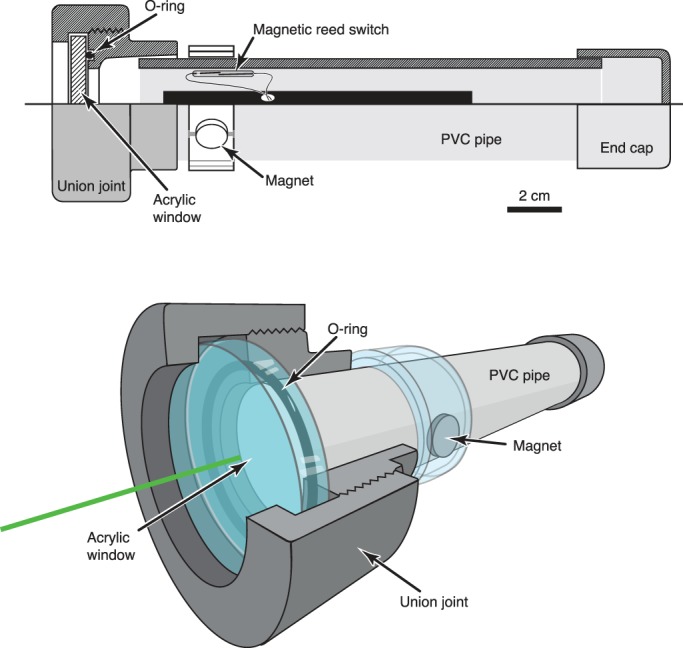
**Underwater housing for laser pointer used for *in-situ* experiments.** Laser was first modified by connecting a magnetic reed switch across leads of the push-button actuator. The housing was built from plumbing hardware, using a PVC union joint which had the pipe fitting opposite the O-ring removed and replaced with a clear acrylic disk. A neodymium magnet outside the tube can be rotated to activate the reed switch inside the tube.

**Fig. 5.**
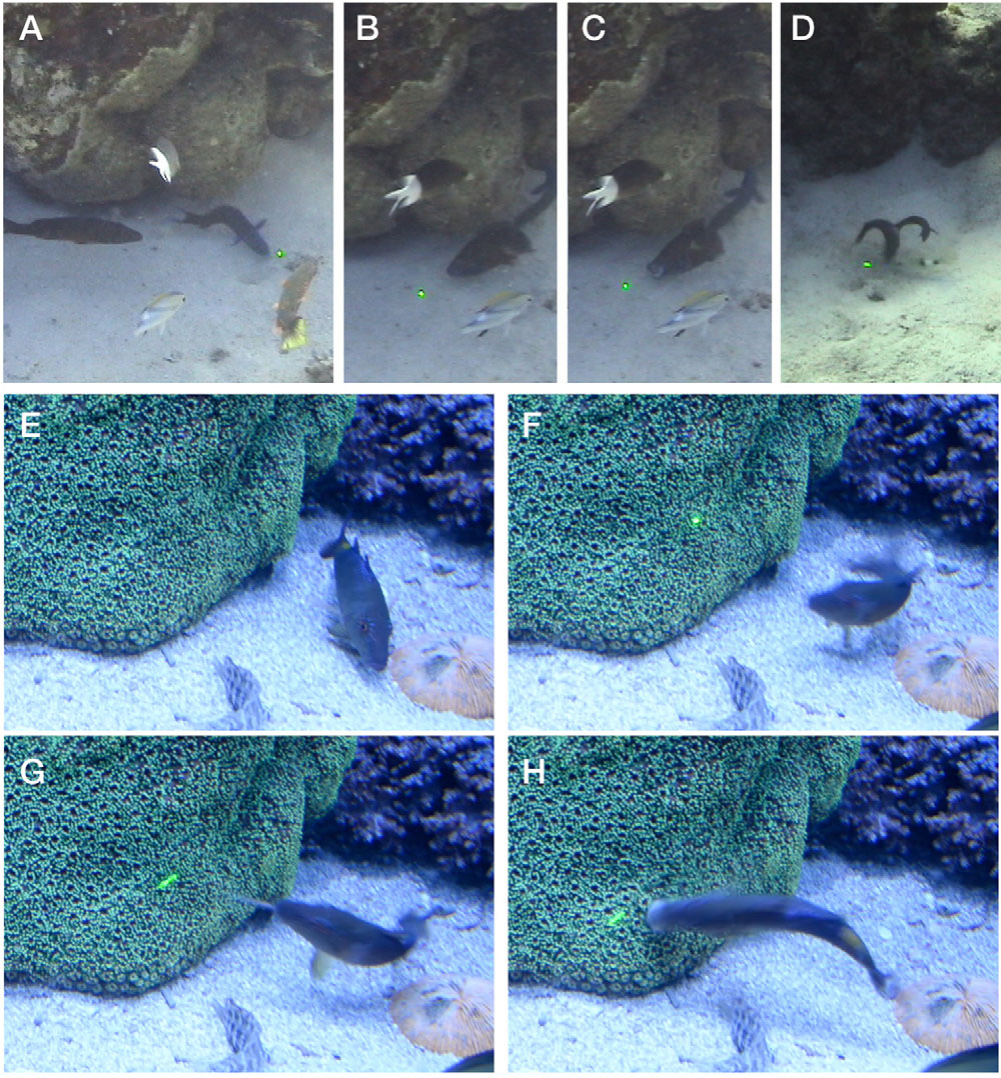
**Frame grabs from video of the green laser deployment underwater.** (A-D) Great Barrier Reef, showing wrasses pursuing the laser across the bottom and biting at it. (E-H) Aquarium footage of a goatfish responding to the appearance of the laser. Interval from E-F is 330 ms, and images G and H are each at 100 ms intervals. In image G, the barbels, laden with taste sensors, are extended to investigate the dot.

To observe the natural responses of fishes to illuminated dots, similar to the view of fluorescent tentacle tips, we constructed an underwater housing for a green laser pointer (Fig. 4), and tested it in several locations as well as in aquaria. During the day, use of the green laser underwater elicited aggressive responses from many fish living on and around coral reefs, especially in sandy patches (Fig. 5; supplementary material Movie 1). Fish made repeated strikes at the point where the laser shone on the substrate. At the Great Barrier Reef, Australia these responses were most pronounced among the benthic species, including blennies (*Enchelyurus* spp.), *Chromis*, lizardfish (*Synodus jaculum*), gleaners such as wrasses (e.g. the black-spot wrasse, blue-headed wrasse), and goatfish (Mullidae, e.g. *Mulloidichthys* sp,, *Parupeneus* sp.). Blennies showed territorial behavior and aggressively chased the spot only for a few centimeters, but wrasses and goatfish pursued the spot persistently for many minutes and across many meters. In Western Papua, many kinds of fish, including young damselfish (*Pomacentrus* sp.), triggerfish (*Balistapus* sp.), wrasses and snappers (*Lutjanus biguttatus*) showed interest and aggression at the dot, including following it across the bottom and biting at it. At night, when the beam was relatively much brighter in the darkness, fish avoided the green beam of light, often swimming rapidly away or even leaping out of the water.

#### Field observations of fluorescence in other organisms

**Fig. 6.**
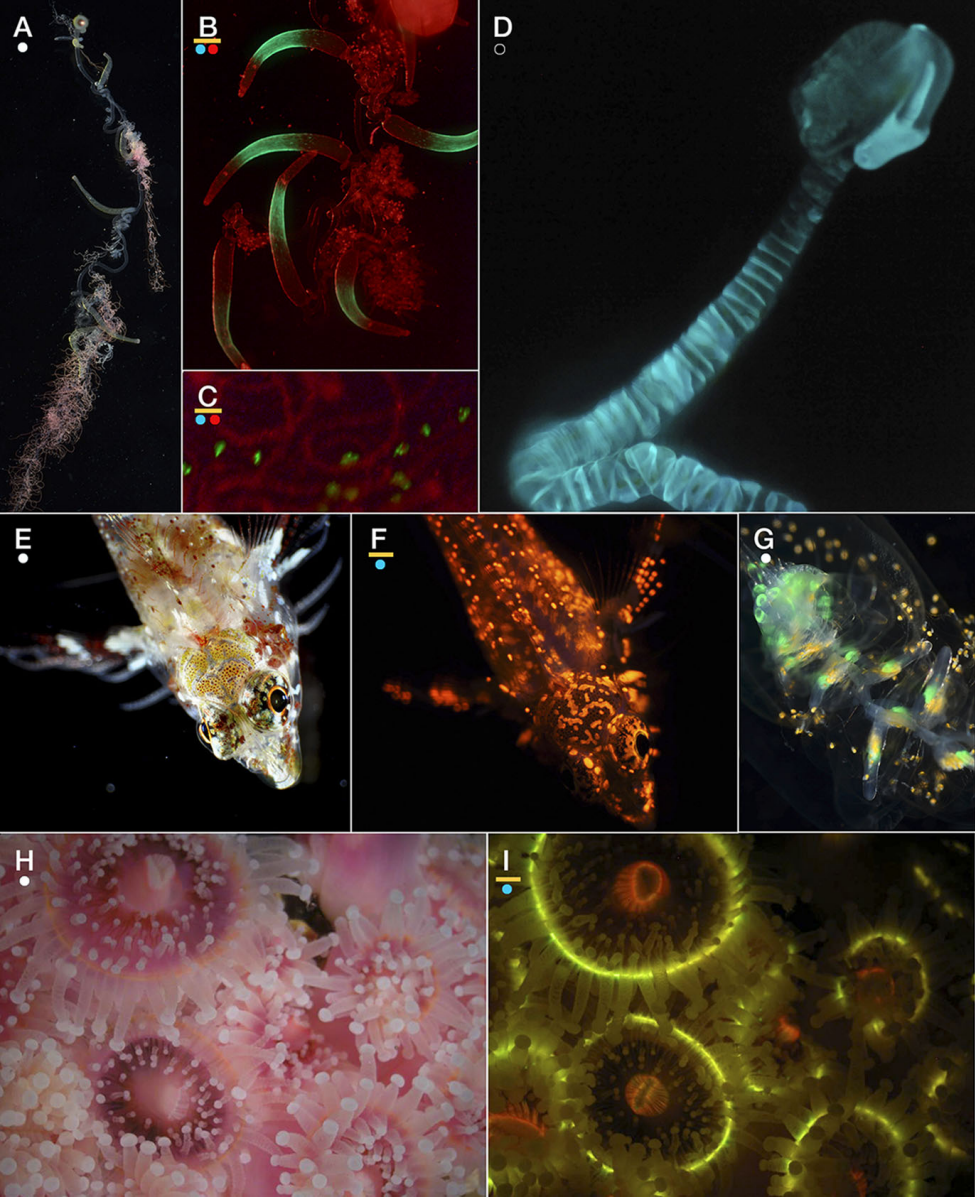

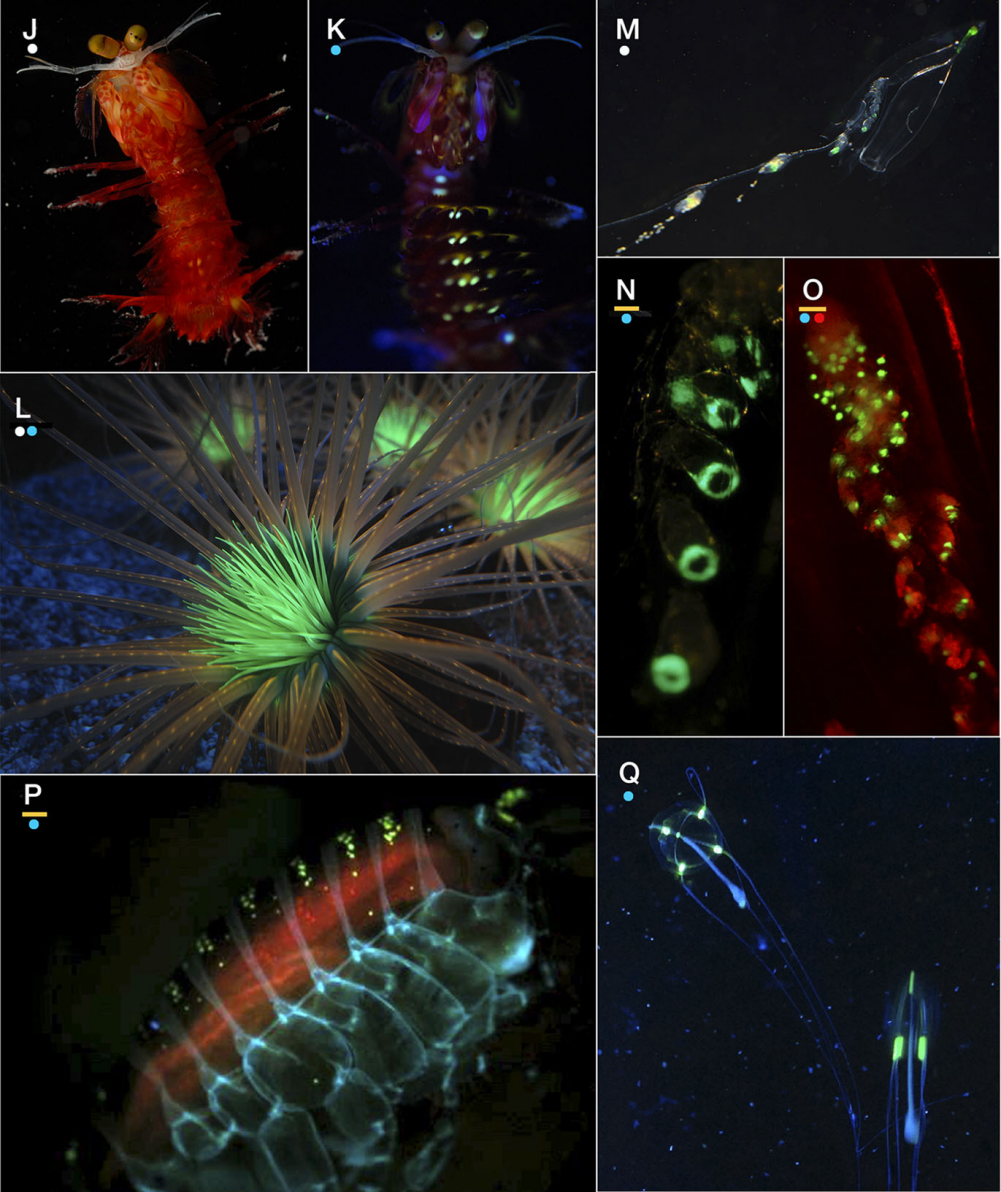
**Examples of species in which fluorescence may be functioning for prey attraction. (**A-C) The siphonophore *Rhizophysa eysenhardti*, showing white light view (A) and green fluorescence (B,C), with red illumination (not fluorescence) to show the rest of the body. (D) Bioluminescence emission of the siphonophore *Rosacea plicata*, with no illumination. Compare with panel G showing the distribution of fluorescence. (E,F) Light and fluorescence of the triplefin blenny *Enneapterygius* sp., a small tropical species with fluorescent skeletal structures. (G) White illuminated photo of *Rosacea* showing the fluorescence near the top of the stem and in the gastrozoids, bright enough to see without special blue excitation or filters. (H,I) White light and fluorescence of the non-symbiotic strawberry anemone *Corynactis californica*, showing the multi-colored fluorescence of its polyps. Scale (width of frame), A: 1.7 cm; B: 1.2 cm; C: 1.3 mm; D: 9.3 cm; E: 8.4 mm; F: 8.6 mm; G: 1.3 cm; H,I: 2.9 cm. (J,K) White light and fluorescence of the mantis shrimp *Gonodactylaceus randalli*. Other mantis shrimp species have strong fluorescence on their second antenna scale. (L) Cerianthid tube anemone under mixed lighting showing prominent fluorescence in central tentacles. (M-O) The siphonophore *Diphyes dispar* under three lighting schemes to show morphology and fluorescence associated gastrozooids (feeding polyps). Even in white light without special excitation (M) the fluorescence is visible, and it is enhanced by blue illumination (N,O). Red light in O is external illumination and not fluorescence. (P) Amphipod *Cyphocaris* showing several types of fluorescence: yellow from bioluminescent structure, blue from chitin, and orange likely from chlorophyll-containing gut contents. (Q) Like the hydromedusa *O. formosus* used in our experiments, *Sarsia tubulosa* has fluorescent structures that are not associated with sites of bioluminescence. Scale (width of frame), J,K: 2.9 cm; L: 9 cm; M: 2.6 cm; N: 8 mm; O: 4.7 mm; P: 11 mm; Q: 6 cm. Dots below panel letters represent color of illumination/excitation used for photos: white, blue, red, or none (bioluminescent light from organism only). Yellow bar above dots indicates when a yellow long-pass barrier filter was used.

Observations of live animals revealed several species with fluorescent structures unrelated to bioluminescence or algal symbioses, some noted for the first time. The implications of this are addressed further in the discussion, and the observations are summarized here. The siphonophores *Rhizophyza eysenhardti* (Fig. 6A-C), *Rosacea plicata* (Fig. 6G), and *Diphyes dispar* (Fig. 6M-O) bore fluorescent spots along the stem of the siphosome or on the tentilla themselves. These spots are quite unlike the nectophore- and bract-associated fluorescence of other siphonophores like *Lilyopsis fluoracantha* (Haddock et al., 2005), in which the fluorescence is considered to be involved in the bioluminescence. While *Rhizophysa* species are to our knowledge non-luminous, *Rosacea* species are bioluminescent; however the pattern of luminescence emission (Fig. 6D) which occurs diffusely across the whole body, does not match the fluorescence patterns (Fig. 6G), which are discrete points on the gastrozooids (feeding polyps).

Fishes, anthozoans, and mantis shrimp also have bright fluorescent structures clearly visible under blue illumination and a long-pass filter (Fig. 6). Unlike corals, the anthozoans *Corynactis californica* (Fig. 6H,I) and cerianthid tube anemones (Fig. 6L) have no algal symbionts, yet they have conspicuous and colorful fluorescence (Schnitzler et al., 2008) around their mouth. Many tropical fish have long-wavelength fluorescence (e.g. Fig. 6E,F) of unknown function. Like *Olindias*, other hydromedusae can also have fluorescence that appears to be unrelated to bioluminescence (Fig. 6Q). Crustaceans including many mantis shrimp (Fig. 6J,K) have bright fluorescent structures, while others may express blue autofluorescence associated with chitinous structures (Fig. 6P) or orange and red fluorescence likely associated with ingestion of chlorophyll (Fig. 6P).

## DISCUSSION

In the ocean, the spectrum of available colors is quickly filtered down to a uniform blue (Johnsen, 2014; Smith and Baker, 1978), and in this largely monochromatic environment, conspicuous coloration cannot be achieved through absorptive pigmentation. Fluorescence provides a mechanism to generate a range of colors only using blue light. Individual animals may have up to six different fluorescent pigments, providing them with an available palette of colors spanning green, yellow, orange, and red (Schnitzler et al., 2008). Objects are most visible when they have high contrast, and Johnsen (2001) showed that over short distances, contrast is greatest at wavelengths displaced from the wavelength of maximum light penetration, dependent on the sensitivity of the perceiver and properties of the water (e.g. Loew and Lythgoe, 1978). In other words, non-blue wavelengths will provide for a highly visible object in a blue-light environment.

Fluorescence can make objects conspicuous by at least two mechanisms: by increasing brightness against the background, and thus contrast for monochromatic receptors, or by increasing the signal received by long-wavelength receptors, against a background signal that is detected by short-wave receptors or filtered by lens pigments. Fluorescence can be more effective than even reflective surfaces at increasing visibility against the background, because when seen from the side, mirrored objects match the radiance of the background (Johnsen and Sosik, 2003).

Many molecules and the biological structures they form are naturally and brightly fluorescent, both in marine animals (e.g. Fig. 6; Michiels et al., 2008) and in terrestrial organisms (Parrots: Hausmann et al., 2003; scorpions, Stachel et al., 1999; butterflies: Vukusic and Hooper, 2005; plants: Kurup et al., 2013). Among the brightest of these natural fluorescent molecules are proteins of the green-fluorescent protein (GFP) superfamily. These have been discovered and cloned from a variety of animals, including sessile and pelagic cnidarians, crustaceans, and even chordates (Wiedenmann, 2004; Deheyn et al., 2007; Shagin et al., 2004). Other examples of autofluorescent molecules and tissues include tryptophan, riboflavin, keratin, chitin, and collagen. Beta-carboline, a tryptophan derivative, and 7-hydroxy-4-methylcoumarin are two small molecules responsible for the fluorescence in the cuticle of scorpions (Stachel et al., 1999).

The roles of such bright colors in marine animals have long been subject of speculation (e.g. Wicksten, 1989). Some cnidarians – in particular octocoral sea pens (*Renilla*) and hydromedusae like *Aequorea, Obelia* (Morin and Reynolds, 1969; Campbell, 1974), and *Phialidium* (=*Clytia*) (Levine and Ward, 1982; Fourrage et al., 2014) – possess GFP-type fluorescent proteins that function to modify the emission wavelength of their bioluminescence system. In fact, the original GFP was purified as a by-product of extraction of the luminescence chemistry from a hydromedusa (Shimomura et al., 1962; Shimomura, 2005). Hard corals and (most) anemones are not bioluminescent, yet they can have a full range of green to red fluorescent proteins (Matz et al., 1999; Alieva et al., 2008). For those groups, most of the previously proposed functions for fluorescent pigments are related to providing a benign light environment for their algal symbionts, either by screening harmful wavelengths (Salih et al., 2000) or by shifting incoming light to wavelengths more favorable to photosynthesis (Schlichter et al., 1994).

However, none of the explanations above apply to fluorescent organisms that are non-bioluminescent, non-visual, and do not have algal symbionts, many of which are shown in Fig. 6. In the ocean, such animals include cerianthid tube anemones (even from deep waters; Vogt et al., 2008), strawberry anemones (Schnitzler et al., 2008), cephalochordates like *Branchiostoma floridae* (Deheyn et al., 2007), mantis shrimp (Mazel et al., 2004), and siphonophores like *Rhizophysa eysenhardti,* discovered herein. The red fluorescence reported from the siphonophore *Physalia physalis* is likely a combination of biliprotein (Herring, 1971) and artifact (Yanagihara, 2003), and is not related to these pigments. For all of these animals, a prey-attraction function like the one demonstrated in our experiments seems to be a likely role for fluorescence. Others have suggested that fluorescence in fish increases with depth, and thus serves a visual, not photoprotective role (Michiels et al., 2008; Meadows et al., 2014). Even corals that do have algal symbionts still rely on zooplankton predation for nutrition and sustenance (Palardy et al., 2006), and crepuscular or lunar feeding cycles would be consistent with the presence of a dim blue light field to excite fluorescence at the times when prey migrate up out of the reef.

### Supernormal stimuli

The experimental portion of this study examined the proposed attraction of juvenile rockfish to the fluorescent lures of the non-luminous, asymbiotic hydromedusa *O. formosus*. The strong and aggressive response in our experiments matches the response that we saw in other fish species during *in situ* observations. Both sets of reactions are consistent with a supernormal stimulus response. A similar hypothesis was proposed for a relative of *O. formosus, O. tenuis*, which vibrated its non-fluorescent tentacles to attract prey (Larson, 1986), but no study has ever quantitatively tested this hypothesis.

Supernormal stimuli are sensory signals – often visual – which are exaggerated versions of the sign stimuli that provoke natural responses [reviewed in Tinbergen, 1948 (the term “supernormal” was not yet in use); Staddon, 1975]. Despite being unrealistic in scale, these stimuli can trigger strong behavioral responses, as has been demonstrated repeatedly in birds, fish, insects, and even humans (Gardner and Wallach, 1965). In the classic experiments, herring gull chicks would respond more strongly to a false beak with an enhanced red spot than to a realistic gull beak (Tinbergen and Perdeck, 1951), and adult birds would preferentially sit on a gigantic cubical “egg”, despite being the wrong color and shape (Tinbergen, 1948). These compulsions can be powerful and lead to maladaptive behaviors. When considering why insects are fatally attracted toward artificial lights, Verheijen (1960) concluded that artificial lighting conditions caused the animal to move to the light source “irrespective of factors which are incompatible with survival.”

In our experiments and during the *in situ* observations, the bright green target (generated by fluorescence or by the laser) created a visually exaggerated signal – a supernormal stimulus – which elicited strong predation behaviors from a variety of fishes. Our results indicate that under natural conditions, predators can exploit unnatural signals to elicit behavior that makes their prey more susceptible to being encountered and captured.

### General use of fluorescence as a prey attractant

The presence of fluorescent pigments in non-luminous, non-symbiotic, and non-visual organisms indicates these have an additional function besides those previously proposed. We documented fluorescent structures in many types of animals which we believe may use these visual signals to attract prey (Fig. 6). Purcell (1981) found a curious result that the non-visual cnidarian predator *Rhizophysa eysenhardti*, a close relative of the Man o’ War *Physalia physalis*, captured its prey of larval fish almost exclusively during the day, even though fish were abundant during the night. *Rhizophysa* species are not bioluminescent. Our observations of green fluorescent spots on the tips of the tentacles and along the gastrozooids in *Rhizophysa* (Fig. 6A-C) suggest that these spots might be conspicuous and attractive to the fish when excited by blue downwelling light during the day. A similar mechanism appears to be at work with *Resomia ornicephala*, which also has fluorescent (non-GFP-type) tentilla (Pugh and Haddock, 2010) to capture euphausiid shrimp, and which has a restricted vertical distribution at a daytime depth around 200 m. At that depth, the dim blue background light provides a predictable source with which to excite fluorescence. *Diphyes dispar* and *Rosacea plicata* are other siphonophores that we found to have fluorescent gastrozooids (Fig. 6G,M-O), unrelated to their bioluminescence which occurs on their bracts and nectophores (swimming bells). We hypothesize that they also can use fluorescence in this manner.

Mazel et al. (2004) describe fluorescent patches in a mantis shrimp (e.g. Fig. 6J,K), and speculate that the fluorescence is used for signaling conspecifics. However, a supplementary video from their study shows a fish that seems to repeatedly approach the fluorescent patches, until it is captured by the shrimp. Pending experimental follow-up, the fish's behavior of directed swimming matches the strike behavior elicited in our laboratory experiments, and we believe that this is also an example of fluorescence serving to create conspicuous lures for attracting prey.

Many of the fluorescent fishes which have recently been catalogued (Fig. 6E,F; Matz et al., 2005; Michiels et al., 2008; Kumagai et al., 2013; Meadows et al., 2014; Sparks et al., 2014; S.H.D.H, personal observation) are ambush predators which sit motionless on the reef and prey on amphipods and other mobile gleaners. Although some of the skin fluorescence in sharks might be a non-functional by-product of the chemistry of structural elements (like the fluorescence of chitin in many arthropods), other patterns may serve ecological functions that have not yet been revealed.

### Fluorescence from the prey's perspective

The advantage to the predators is obvious, but why would prey be attracted to bright fluorescent colors? Chlorophyll fluorescence is used by plant physiologists and ecophysiologists to gain information about photochemistry and the efficiency of photosynthesis (Maxwell and Johnson, 2000). Fluorescence has even been suggested to enhance prey-trapping abilities of carnivorous plants (Kurup et al., 2013). Algal tufts, films, and mats can show fluorescent red on the reef, and chlorophyll can also fluoresce in the guts of animals (Fig. 6P). Herbivorous prey may be attempting to find algae and be attracted by long-wavelength fluorescent pigment that would normally indicate the presence of chlorophyll, while carnivorous prey could be searching for gut fluorescence or any high-contrast target against the uniform background.

Many factors affect the evolution of visual sensitivities and behaviors among organisms. Cummings (2007) showed that vision and color sensitivity in some kelp forest fishes evolved primarily for foraging and habitat; mating coloration was secondary adapted to the visual systems that had evolved within those constraints. In lab experiments, Mussi et al. (2005) showed that fish were more attracted to prey that were bright against a dark background, consistent with a fluorescent signal. In rank order the visual factors that contribute to prey selection are color>movement>shape>size (Ibrahim and Huntingford, 1989), although other studies found that movement≫color (Kislalioglu and Gibson, 1976). Either scenario supports the attractive properties of a brightly colored and motile element such as the fluorescent tentilla borne by *O. formosus* and *Rhizophysa eysenhardti*.

### Conclusions

Fluorescence is likely to serve many roles for marine animals, and in many cases its presence may not have ecological consequences at all. However, our experimental and observational evidence from the laboratory and field, supports the hypothesis that prey attraction may be a primary ecological function for fluorescent pigments in general, and GFP in particular.

## MATERIALS AND METHODS

### Attraction experiments

**Fig. 7.**
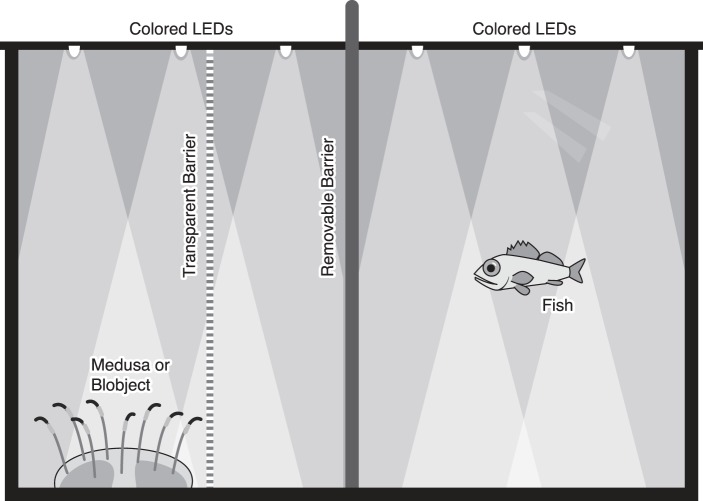
**Experiments were conducted in a custom-built aquarium with opaque sides and transparent front.** A clear barrier was fixed in place between the medusa and the fish, and an opaque barrier could be inserted between the fish and the target. The two opaque lids over the top each contained four colored LEDs, which could be changed out for the trials.

Predators (medusae) and prey (fish) were placed in custom-built acrylic tanks (33.75 cm×18.75 cm×22.5 cm). The back and sides of the tanks were opaque black, and the front was transparent. The tanks had two black lids, each with 4 holes for embedding light-emitting diodes (LEDs). LEDs of the treatment color (blue, yellow, or white) were evenly distributed to uniformly illuminate the tank (Fig. 7). This mimicked a natural setting (both fish and jellyfish illuminated), and ensured the observer could see the fish at all times.

The predator in each trial was a specimen of the hydromedusa *O. formosus* (Fig. 1), obtained on loan from the Monterey Bay Aquarium and originating from Japan. Medusae were 5 to 9 cm in diameter. The potential prey were juvenile eastern Pacific kelp rockfish *Sebastes atrovirens* and black-and-yellow rockfish *Sebastes chrysomelas*, part of the so-called KGB species complex. Fish were obtained from Shane Anderson at the University of California, Santa Barbara, and returned intact after the experiments. *Olindias* species are known to eat fish, but the exact prey species is unknown. Because of similar habitat characteristics and their visual feeding habits, these juvenile *Sebastes* were considered a realistic substitute for natural prey.

Due to ethical consideration and scarcity of experimental organisms, the medusae were not actually allowed to feed on the fish. Instead, a clear barrier was inserted into the tank, so that fish could see the predator, but not come into contact. The barrier was put into place so that the tank was divided into two volumes with a ratio of 3:1. The larger volume was where the fish was placed, and the smaller side contained the live medusa or a plastic medusa mimic as a control.

Before each trial, a black barrier was placed in the middle of the tank, so the fish could not see the medusa. Before the first trial, the medusa was placed in the tank, and the animals were allowed to acclimate for 30 min at the beginning of each day of trials. With the medusa and a fish placed into each side of the tank, the LEDs were turned on. When the trial was to begin, the black barrier was slowly removed, and the behavior of the fish was observed. The trials were 15 min long and were performed under dark-room conditions. The amount of time the fish spent in the half of its partition of the tank adjacent to the medusa or blobject (Fig. 7) was measured, along with the number of times the fish attacked the clear barrier. An attack was defined to be a fast burst of swimming directed toward the medusa or blobject behind the clear barrier; in most cases the mouth was open during these motions. The attacking motion was such that if the clear barrier had not been in place, each attack made by a fish would have resulted in actual contact with the medusa, and most likely a capture.

Tests under each lighting scheme were conducted under three prey conditions: none (no object), medusa (*O. formosus* present), and blobject (decoy medusa present). The no object condition (nothing present behind the barrier) was used to record the fish's typical behavior in the experimental tank without the general shape of the medusa present and without any tentacle-specific signal. For tests with the live *O. formosus* present, the medusa was placed into its side of the tank and allowed to settle into its typical ‘fishing’ behavior (not swimming, oriented bell-up while sitting on bottom, with tentacles outstretched) before the test began. During this period, the fish could not see the medusa because the opaque barrier between them had not yet been removed. At the beginning of a trial, the fish would often remain motionless in one area of the tank. To account for this, experimental time did not begin until the fish crossed the halfway mark of the tank to signal active swimming.

Seventeen trials of each combination of lights (three states) and targets (three states) were conducted, beginning with blue light and an empty tank, and finishing with white light and blobject. Ten different fish were used in the trials and fish species is not treated as a factor because they are expected to behave the same. Each individual fish experienced a different combination of treatments without repetition, and with each fish having its first exposure to a different initial condition. All the time measurements and counts of attacks were then averaged over the multiple trials and error bars reported are the standard error of the mean (box height) and 95% confidence interval (whisker line).

### Statistical methods

Analyses were carried out using R (R Development Core Team, 2008) version 3.1.2. Raw data and full analysis commands are included in the supplementary materials Data S1 and S2. Briefly, experiments were analyzed under a Type-I Anova with two fixed factors and equal replication, using the aov() function in R: aov(Attacks∼Color×Jelly, data=d.f.). Plots were generated using ggplot2 (Wickham, 2009).

### Spectra of light sources

The spectra of the blue, yellow, and white LEDs were measured using an OceanOptics USB2000 spectrometer through a UV-transparent fiber optic cable. The emission peak of the *O. formosus* fluorescent protein (FP) was measured in seawater from whole tissue tentacle tips removed from the bell of the medusa*.* The FP was excited using a blue LED with maximum output at 479 nm and a violet LED (405 nm), and measured using USB2000 spectrometer. Absorbance spectra and the excitation spectra of the fluorescent protein and the pink chromoprotein were measured with a Shimadzu UV-1650PC spectrophotometer.

### *In situ* behavior of other animals

For construction of the underwater laser pointer, the push-button switch was rewired to a magnetically activated reed switch, and together they were placed inside a PVC tube. A PVC union joint was modified by removal of one of the elements, leaving a face-sealed o-ring surface. This was fitted to the tube and clear acrylic pane was inserted, allowing the laser light to be emitted. A neodymium magnet was embedded into a sliding ring on the outside of the tube, which could be rotated adjacent to the reed switch to turn the laser on while underwater. More recently, underwater laser pointers have become commercially available.

Fish responses were filmed with underwater video cameras during SCUBA dives in reefs off the coast of Australia, Papua, and Hawaii, and observations were made both during the day and nighttime.

### Morphological observations

Planktonic and benthic predators were collected in glass jars during blue-water SCUBA dives (Haddock and Heine, 2005). Their fluorescence, morphology, and behavior were observed using a Nikon SMZ-1500 microscope with 470 nm excitation and a long-pass (>500 nm) filter set or yellow acrylic filter (TAP Plastics Transparent Dark Yellow, acting as a long-pass element). For non-microscope shots, macro photographs were mainly taken with Nikon D600 and D90 cameras with 60 mm macro lens, and manual settings, with white-balance set to daylight or ‘fluorescent light’ mode. Specimens of particular interest were the siphonophores *Diphyes dispar* and *Rhizophysa eysenhardti* from the Gulf of California, Mexico, and the mantis shrimp *Gonodactylaceus randalli* from Moorea, French Polynesia.

## Supplementary Material

Supplementary information
