# Supplementary material for: Fluorescent proteins function as a prey attractant: experimental evidence from the hydromedusa Olindias formosus and other marine organisms
Source: Biol Open. 2015 Jul 31;4(9):1094–104. doi: 10.1242/bio.012138 (PMC4582119; doi:10.1242/bio.012138)
Supplement: Supplementary information [file supp_4_9_1094__index.html]

Fluorescent proteins function as a prey attractant: experimental evidence from the hydromedusa Olindias formosus and other marine organisms — Supplementary information 

# Fluorescent proteins function as a prey attractant: experimental evidence from the hydromedusa *Olindias formosus* and other marine organisms

## BIO012138 Supplementary information

**Files in this Data Supplement:**

- Supplementary information
