# Supplementary material for: Fluorescent proteins function as a prey attractant: experimental evidence from the hydromedusa Olindias formosus and other marine organisms
Source: Biol Open. 2015 Jul 31;4(9):1094–104. doi: 10.1242/bio.012138 (PMC4582119; doi:10.1242/bio.012138)
Supplement: Supplementary information [file supp_bio.012138_BIO012138supp.pdf]

Supplementary material Movie

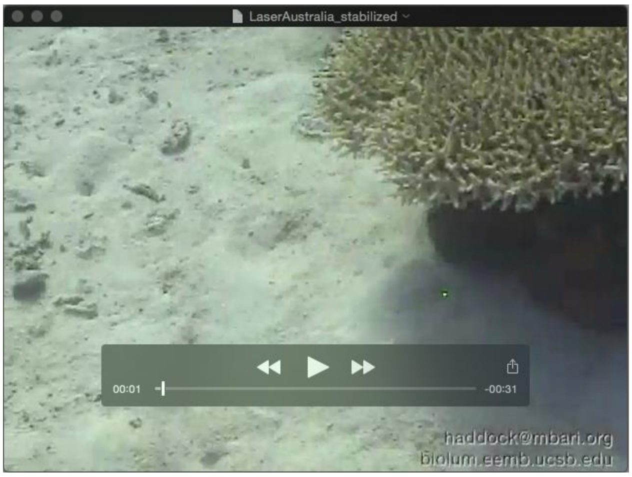

Supplementary material data S1.

Click here to Download data S1

Olindias Fluorescence attraction

Supplementary information file for: Haddock and Dunn. Fluorescent proteins function as a prey attractant: Experimental evidence from the hydromedusa Olindias formosus and other marine organisms.

Analysis of the effect of Light Color and Target type (Jellyfish with Fluorescent tentacles) on fish predation.

Type I ANOVA

Two fixed (non-random) balanced factors mean that a type I ANOVA is appropriate. The only concerns are: * if the sample sizes are big enough because there are difficulties with CI when n<30 * whether parametric stats can be used with data distributed as they are.

Histogram of attack numbers

```
ggplot(data=df,aes(x=Attacks)) + geom_histogram(aes(fill=Color),binwidth = 5) +
    facet_grid(Color~Jelly) +
    scale_fill_manual(values=c("lightblue","white","lightyellow")) +
    labs(title="Distribution of Attack freqs")
```

Analysis of variance results from *Type I*

Fixed factor with equal replication

Summary of Attack Results

Attacks on jelly with different trials

```
df_aov=aov(Attacks~Color*Jelly,data=df)  # do the anova
summary(df_aov)    # show table
```

```
##              Df Sum Sq Mean Sq F value   Pr(>F)
## Color         2  236.6  118.29   8.436 0.000343 ***
## Jelly         2  187.1   93.55   6.672 0.001695 **
## Color:Jelly   4  448.7  112.17   7.999 7.49e-06 ***
## Residuals   144 2019.2   14.02
## ---
## Signif. codes:  0 '***' 0.001 '**' 0.01 '*' 0.05 '.' 0.1 ' ' 1
```

Biology Open • Supplementary information

```
print(model.tables(df_aov,"means"),digits=3) # show means and numbers
```

```
## Tables of means
## Grand mean
##
## 1.764706
##
##  Color
## Color
##   Blue  White Yellow
##   3.49   0.61   1.20
##
##  Jelly
## Jelly
## Blob None  Yes
## 0.57 1.49 3.24
##
##  Color:Jelly
##         Jelly
## Color    Blob None Yes
##   Blue   0.88 1.24 8.35
##   White  0.41 1.18 0.24
##   Yellow 0.41 2.06 1.12
```

Summary of Time Spent Results

Time spent in jelly half of tank (indicating interest)

```
df_aov.Fraction=aov(FractionTime~Color*Jelly,data=df)  # do the anova
summary(df_aov.Fraction)    # show table
```

```
##              Df Sum Sq Mean Sq F value Pr(>F)
## Color         2  0.042 0.02081   0.381 0.6837
## Jelly         2  0.066 0.03312   0.607 0.5465
## Color:Jelly   4  0.564 0.14110   2.585 0.0395 *
## Residuals   144  7.860 0.05459
## ---
## Signif. codes:  0 '***' 0.001 '**' 0.01 '*' 0.05 '.' 0.1 ' ' 1
```

Calculate statistics per factor

Note confidence interval, etc not reliable when n<30

Biology Open • Supplementary information

Interactions for Attack number

```
Int.Attack = ddply(df,.(Color,Jelly),summarise,
                                    M=mean(Attacks),SD=sd(Attacks),
                                    SE=(sd(Attacks)/sqrt(length(Attacks)-1)),
                                    N=length(Attacks))

conf_interval=.95
ciMult <- qt(conf_interval/2 + .5, Int.Attack$N-1)
Int.Attack$CI <- Int.Attack$SE * ciMult

Int.Attack
```

```
##    Color Jelly         M        SD        SE  N        CI
## 1   Blue  Blob 0.8823529 1.1114379 0.2778595 17 0.5890358
## 2   Blue  None 1.2352941 1.6404806 0.4101201 17 0.8694159
## 3   Blue   Yes 8.3529412 9.5127623 2.3781906 17 5.0415388
## 4  White  Blob 0.4117647 0.8702603 0.2175651 17 0.4612173
## 5  White  None 1.1764706 2.0986690 0.5246673 17 1.1122449
## 6  White   Yes 0.2352941 0.5622957 0.1405739 17 0.2980034
## 7 Yellow  Blob 0.4117647 0.7122871 0.1780718 17 0.3774953
## 8 Yellow  None 2.0588235 3.9285905 0.9821476 17 2.0820600
## 9 Yellow   Yes 1.1176471 3.2187411 0.8046853 17 1.7058566
```

Plot interaction with standard error bars

```
ggplot(Int.Attack, aes(x=factor(Jelly), y=Attacks, colour = Color)) +
geom_line(data = Int.Attack, aes(y = M, group = Color)) +
geom_point(data=Int.Attack, aes(y=M, group=Color),size=3,color="black") +
geom_linerange(data=Int.Attack, aes(y=M, ymax = M+SE, ymin = M-SE, group=Color), size=10,width = 5,alpha=0.3) +
geom_errorbar(data=Int.Attack, aes(y=M, ymax = M+CI, ymin = M-CI, group=Color), width=.1)+
scale_colour_manual(values=c("#00AACC","#888888","#CCCC00")) +
theme_minimal() + labs(title="Attacks by factor with SE (bar) and 95% CI (whisker)")
```

Execution of this manuscript

This manuscript can be recompiled from the source with the following bash commands:

```
Rscript -e "require(knitr); require(markdown); knit('S1_Olindias_Data_Markdown.Rmd')"
pandoc S1_Olindias_Data_Markdown.md -o S1_Olindias_Data_Markdown.pdf
```

This requires:

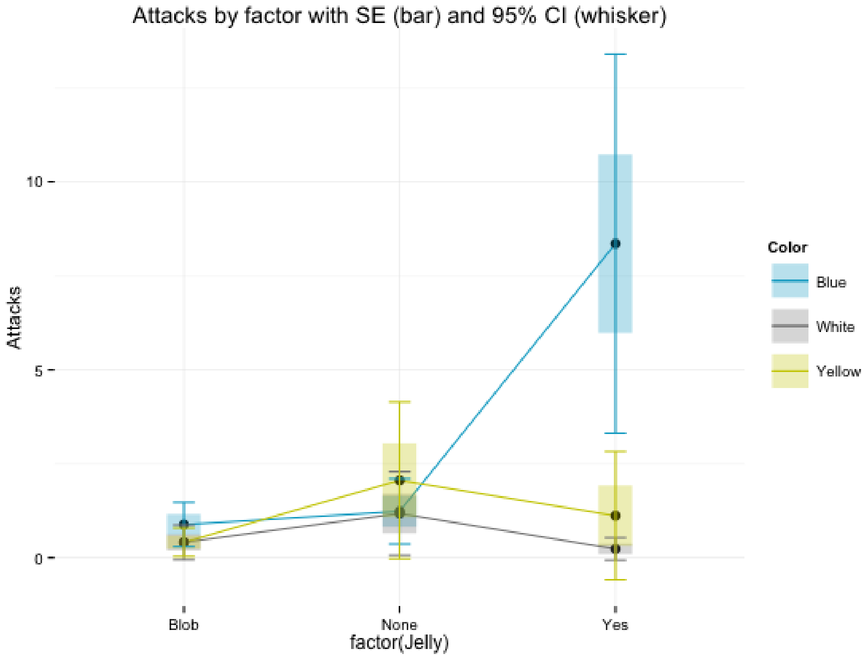

Figure 1: Box Plots of Number of Attacks

Biology Open • Supplementary information

- R (http://www.r-project.org)
- The R libraries ggplot2, plyr, knitr
- pandoc (http://pandoc.org)

This manuscript was computed on Mon Jun 01 20:18:12 2015 with the following R package versions.

```
R version 3.1.2 (2014-10-31)
Platform: x86_64-apple-darwin13.4.0 (64-bit)

locale:
[1] en_US.UTF-8/en_US.UTF-8/en_US.UTF-8/C/en_US.UTF-8/en_US.UTF-8

attached base packages:
[1] stats     graphics  grDevices utils     datasets  methods   base

other attached packages:
[1] plyr_1.8.2    ggplot2_1.0.1 knitr_1.10.5

loaded via a namespace (and not attached):
 [1] colorspace_1.2-4 digest_0.6.4     evaluate_0.7     formatR_1.0
 [5] grid_3.1.2       gtable_0.1.2     htmltools_0.2.6  labeling_0.3
 [9] MASS_7.3-35      munsell_0.4.2    proto_0.3-10     Rcpp_0.11.5
[13] reshape2_1.4.1   rmarkdown_0.5.1  scales_0.2.4     stringr_0.6.2
[17] tools_3.1.2      yaml_2.1.13
```
